# A Case of Testicular Tumor Presenting as Acute Scrotum

**DOI:** 10.7759/cureus.44185

**Published:** 2023-08-27

**Authors:** Zainab A Alturaiki, Ahmad A Alnasser, Ali M Alabandi

**Affiliations:** 1 Department of Urology, Dammam Medical Complex, Dammam, SAU

**Keywords:** testicular torsion, acute scrotum, atypical presentation, germ cell tumor, testicular tumor

## Abstract

Germ cell tumors (GCTs) are the most common testicular malignancies in men. GCT typically presents as a painless nodule or swelling of the testicles with an atypical presentation such as heaviness or dull ache of the scrotum and/or the lower abdomen. Herein, we report a rare case of a testicular tumor presenting as an acute scrotum. This is a case of a 25-year-old male with no significant medical history; he presented to the emergency department with an acute scrotum for less than one day that was highly suspicious for testicular torsion. Surgical exploration and histopathology investigation revealed that this was a case of mixed GCTs.

## Introduction

Germ cell tumors (GCTs) are the most common malignancies of the testicles in men between the ages of 15 and 44 [1]. Histologically, GCTs are divided into seminomas and nonseminomas. Furthermore, nonseminomas are classified into five types -embryonal carcinoma, yolk sac tumor, polyembryoma, choriocarcinoma, and teratoma [1,2]. More than 50% of GCTs contain more than one type of tumor (i.e., mixed GCTs).

GCT usually presents as a painless nodule or swelling of the testicle. In one-third of the cases, the patient might present with heaviness or dull ache of the scrotum or lower abdomen [1]. In <10% of cases, the symptoms of the metastatic disease might be the first presentation including back pain, gastrointestinal (GI) problems, and cough. In rare cases, low food intake, weight loss, and tetraparesis due to spinal cord compression have been reported [2-4].

In typical situations, tumor markers (such as alpha-fetoprotein (AFP), beta-subunit of human chorionic gonadotropin (ß-hCG), and ultrasound imaging) are enough to confirm the diagnosis of a malignant testicular tumor [2]. The management of malignant testicular tumors usually involves surgery and chemotherapy, which is very effective. Herein, we report a rare case of testicular tumor presenting as a case of acute scrotum.

## Case presentation

A 25-year-old single male with no known medical conditions was referred from a rural hospital as a case of left-testicular torsion for organ-saving intervention with a testicular ultrasound (US) report showing no flow in the left testicle. The patient presented to our emergency department with the main complaint of sudden onset severe left-testicular pain and scrotal swelling for 24 hours. His pain was not relieved by analgesics and antibiotics given in the rural hospital, and it was not radiating. Furthermore, he had no history of trauma, urethral discharge, fever, or sexual contact. Physical examination showed that the patient was conscious, alert, oriented, and ill-looking. He was afebrile and his vital signs were within normal limits. Local examination revealed that the right testicle was normal in size, position, and texture without tenderness. The left testicle was swollen, large, and hard with clear tenderness. The position of the testicle could not be appreciated, and there was a negative cremasteric reflex.

**Figure 1:**
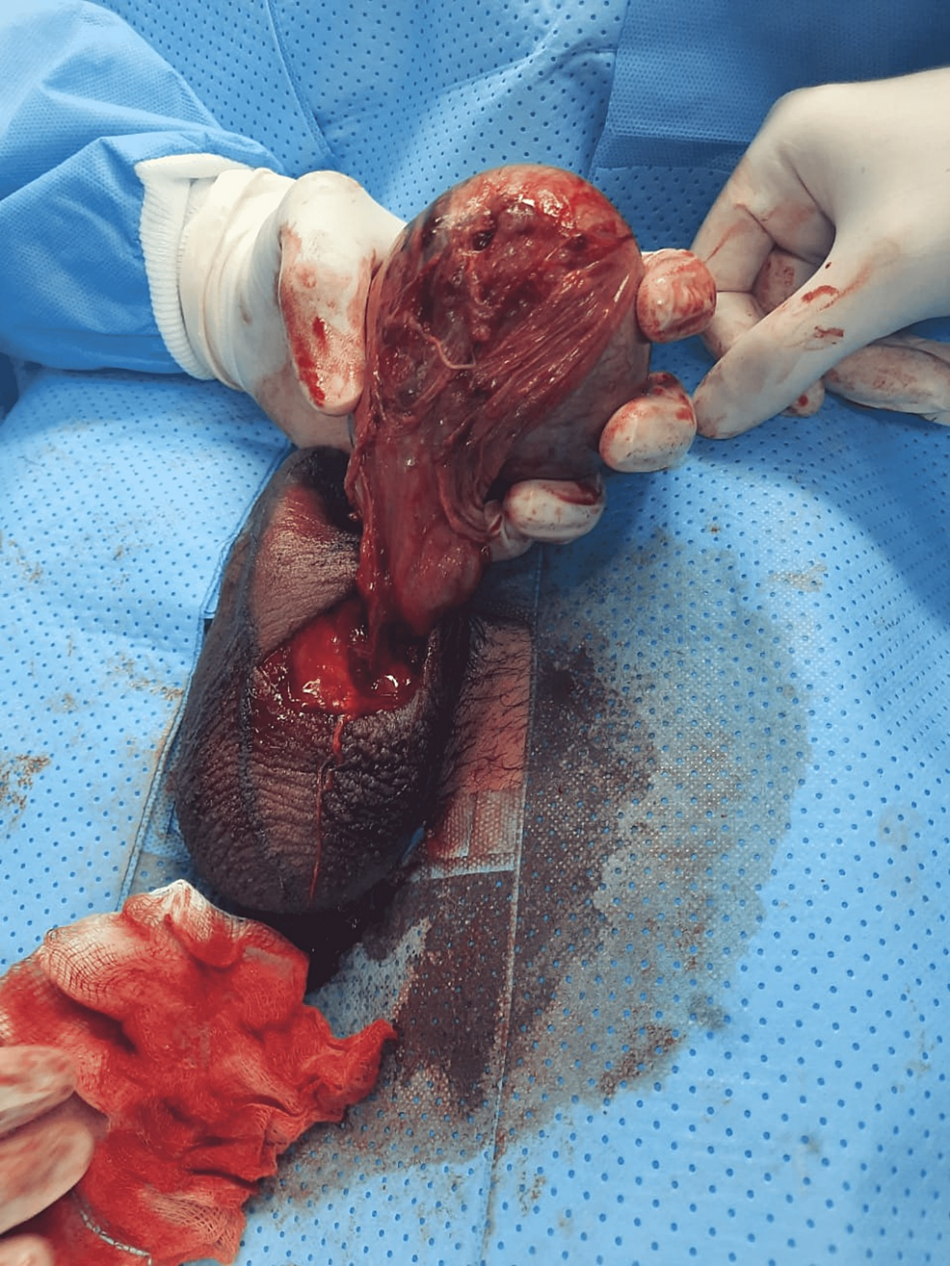
Gross image of the testicle during scrotum exploration showing significant adhesions.

**Figure 2:**
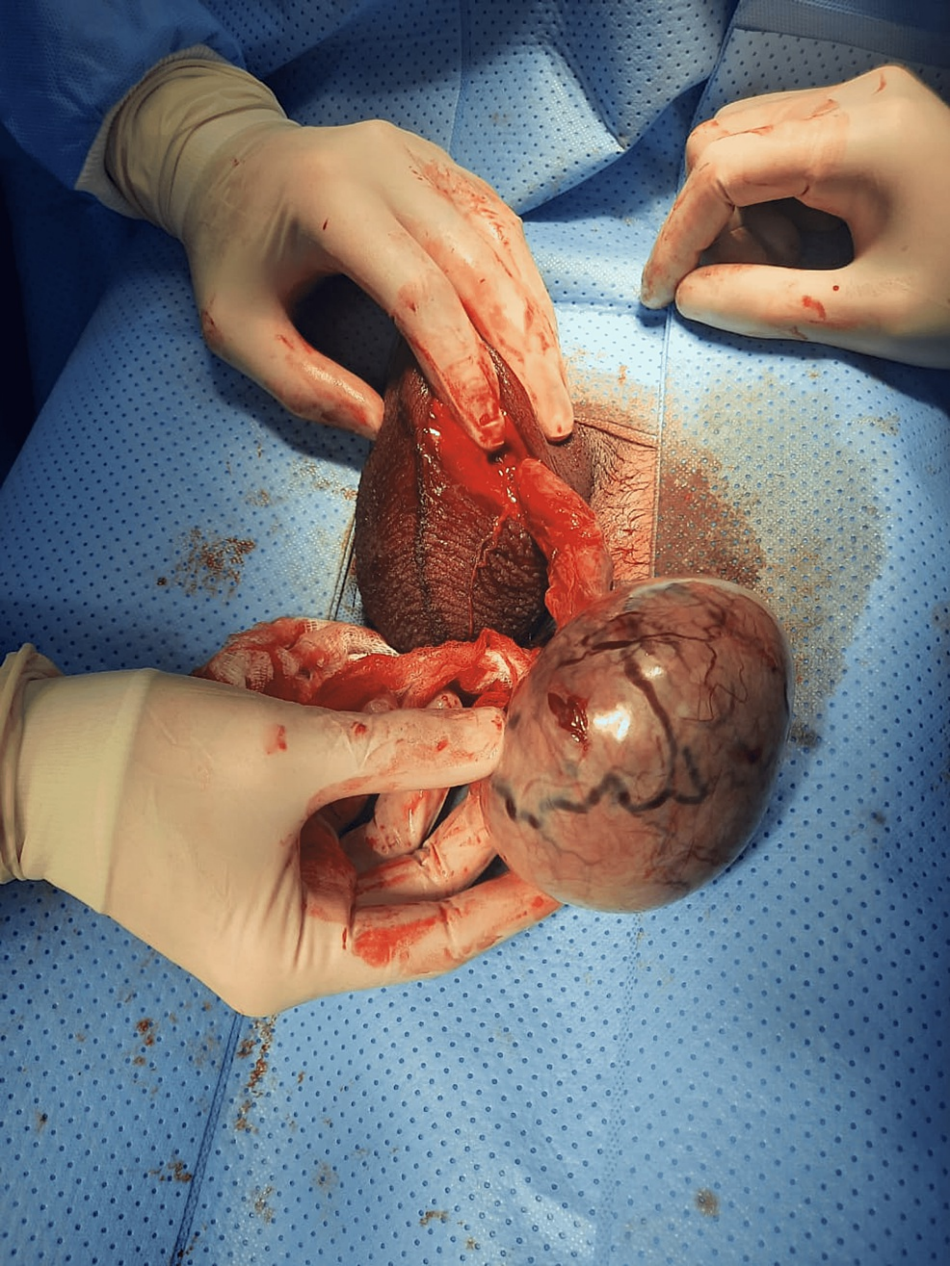
Gross image of the testicle after removal of the adhesions.

Laboratory investigations revealed a white blood cell count (WBC) of 16.5 x 10^9/L, which is elevated above the normal range of 3 x 10^9/L to 10 x 10^9/L, hemoglobin, renal panel, and liver panel were within normal ranges. We decided on a scrotal exploration due to a high suspicion of testicular torsion by clinical assessment. Intraoperatively, torsion was not found; however, the left testicle was severely inflamed with significant adhesions (Figure 1). These adhesions were removed (Figure 2). Tunica vaginalis was opened and had a small reactive clear fluid hydrocele. Furthermore, a small opening in the tunica albuginea was created, which revealed severely inflamed necrotic tissue. Thus, we decided to perform a left orchiectomy, and the left testicle was sent to the laboratory for histopathological analysis. The operation went smoothly, and the patient was doing well postoperatively. His wound was in good status. Upon discharge from our hospital, a blood sample was taken to measure tumor markers. These markers can remain in the blood for a few days after surgery because they are still in their half-life period, so they were sent to the laboratory to be analyzed.

After two weeks, he presented to the outpatient department in our hospital in good health, and his wound healed well. Tumor makers revealed an alpha-fetoprotein (AFP) level of 37.59 ug/L, which is significantly above the normal range of 0 to 7 ug/L. and a beta-human chorionic gonadotropin (beta-hCG) level of 0.407 mlU/mL, which is below the normal range of 0.5 mlU/mL to 2.67 mlU/mL. The histopathological analysis revealed an enlarged testis, gray-tan in color, glistening surface, measuring 11 x 7.5 x 7 cm. Weighing 280 gm. It was made up of mixed germ cell tumors (~60% embryonal carcinoma, ~35% yolk sac tumor, and ~5% teratoma). Attached spermatic cord, measuring 8.5 x 0.7 cm. Small nodule identified on the surface (1.5 x 1 x 0.5 cm). Sectioning show soft gray white variegated appearance mass with necrosis replacing the whole testis. Free epididymis measuring 5 x 1 x 0.7 cm. The tumor had invaded the tunica albuginea and reached the tunica vaginalis. The tumor was classified as T2N0M0. All margins were negative for the tumor. Immunohistochemical stains were not available at our institution at that time. Lastly, the patient was referred to a higher center for urologic oncology services for continuation of treatment as it was not available in our center.

## Discussion

According to the literature, testicular tumors rarely present as acute scrotum. The incidence of this presentation is 10 cases per 100,000 men with testicular tumors and 4.5 cases per 100,000 children or men under the age of 25 [5,6]. The case of acute scrotum typically raises the suspicion of some usual differential diagnoses such as testicular torsion, epididymitis, orchitis, Fournier’s gangrene, testicular rupture, varicocele, or hydrocele. Testicular tumors are not usually considered at the top of the differential diagnosis [1].

Most GCTs are discovered incidentally; the rest are usually diagnosed in cases where testicular mass is the main complaint. In rare cases, the patient’s main complaint is cachexia and anorexia [2]. In another case, the main complaint was tetraparesis for 24 hours due to spinal cord compression [7]. In both cases, the symptoms were believed to be consequences of the far metastasis of an advanced-stage testicular nonseminomatous GCT [3]. In another reported case of a young male patient who presented to the emergency department with an acute scrotum, exploration was performed due to the inability to rule out testicular torsion subcapsular arterial bleeding, and a small nonseminomatous GCT was discovered [4]. The last reported case was from China of a 25-year-old man with an acute scrotum; it was eventually diagnosed as torsion of a testicular tumor [8]. Although all reported cases were of patients with an acute scrotum (diagnosed as a testicular tumor), none reported the tumor as a direct cause of the testicular pain.

In cases of acute scrotum, initial ultrasonography may help distinguish between hemorrhage, inflammation, and testicular mass, given that 50% of pathologic findings are infectious diseases and only 9% classify as tumors [9]. In our case, the patient’s only complaint was acute testicular pain for one day. After surgical exploration, the diagnosis of GCT was confirmed. We discovered no mass effect or low blood perfusion to explain the direct cause of the acute pain.

Diagnostic evaluation of testicular tumor starts with scrotal ultrasound, followed by other radiographic imaging (as needed) and serum tumor markers. It is then confirmed by radical orchiectomy and histopathological studies. Scrotal ultrasound has high accuracy in differentiating between intrinsic and extrinsic testicular lesions, followed by a high-resolution CT scan of the abdomen and pelvis and chest if metastasis is suspected [10].

The three tumor markers with a clear role in the diagnoses and management of testicular GCTs are the beta subunit of human chorionic gonadotropin (beta-hCG), alpha-fetoprotein (AFP), and lactate dehydrogenase (LDH). They are usually found elevated in 80% to 85% of patients with nonseminomatous GCTs. Those tumor markers can be used as supportive evidence of the diagnosis of GCT and are useful to measure the prognosis as well as the response to the treatment of such a tumor. They cannot be used to diagnose a CTG tumor without histopathological confirmation [11]. Regarding the treatment, orchiectomy is still considered the standard of care for local treatment and definitive pathologic diagnosis [12].

The histological features of the tumor in our case were consistent with a mixed germ cell tumor. Embryonal cells were the most common cell type, accounting for 60% of the tumor. These cells are large and pleomorphic, with irregular nuclear borders. The cytoplasm is homogeneously amphophilic or vacuolated. The nuclei come with irregular nuclear membranes and one or more prominent nucleoli. Mitotic figures are frequent. Yolk sac tumor cells are the second most common cell type in our case, accounting for 35% of the tumor. These cells are arranged in anastomosing glandular and ductal structures. The nuclei are large and irregularly shaped, with variable chromatin distribution and one or more prominent nucleoli. The cytoplasm is usually vacuolated. Teratoma cells are the least common cell type in our case, accounting for 5% of the tumor [13]. These cells are more mature and show recognizable elements of more than one germ layer. Those histological features of the tumor confirmed the diagnosis of mixed germ cell tumor

## Conclusions

In conclusion, the diagnosis of testicular tumors should be considered in the presentation of acute scrotum alone even if there are no other symptoms; this pain could be the first clue to the discovery of such a tumor. Furthermore, typical signs and symptoms such as scrotal swelling, weight loss, and night sweating, might not be present.
